# Optimization of the Maritime Signaling System in the Lagoon of Venice †

**DOI:** 10.3390/s19051216

**Published:** 2019-03-10

**Authors:** Fabiana Di Ciaccio, Paolo Menegazzo, Salvatore Troisi

**Affiliations:** 1 Department of Sciences and Technologies, Parthenope University of Naples, 80143 Naples, Italy; salvatore.troisi@uniparthenope.it; 2 Strategic Planning, Port Authority of Venice, 30123 Venice, Italy; paolo.menegazzo@port.venice.it

**Keywords:** virtual AtoN, AIS, AtoN, ECDIS, e-navigation, safety, route planning, visibility

## Abstract

Aids to Navigation (AtoN) are auxiliary devices intended to support maritime navigation. They include both traditional signals (e.g., buoys and lights) and electronic aids, as for example those transmitted to ships through automatic tracking systems. In both cases, international organizations together with local authorities define technical specifications and standards on their use. Work still being finalized in the Venetian Lagoon made it necessary an assessment of the existing signaling system to guarantee the maximum level of safety in the waterways. Considering the severe atmospheric conditions to which the Lagoon is frequently subjected and the bathymetry restrictions affecting the navigation, an alternative aid system has been formalized for the first time in Italy. It is based on electronic and identification devices employed to virtualize the AtoN that will not be located at sea but only remotely identified by their coded messages, thus guaranteeing the continuity of port operations in any visibility conditions. This paper presents the procedures followed to reach a solution in line with the safety and efficiency standards given for the AtoN systems, considering position and luminous characteristics of physical signals in the first case, theoretical and statistical studies on Virtual AIS AtoN placement in the second case.

## 1. Introduction

The International Maritime Organization (IMO) issues standards on security, safety and environmental performance of shipping, encouraging innovation and efficiency. IMO works, among the others, with the International Association of Marine Aids to Navigation and Lighthouse Authorities (IALA), a non-profit technical organization which aims to harmonize Aids to Navigation (AtoN) worldwide considering the needs of mariners and authorities as well as the technological development. To assure the maximum level of safety and efficiency of maritime navigation, IALA publishes recommendations and guidelines on the use of auxiliary devices that improve maritime operations, such as Vessel Traffic Services (VTS), Automatic Identification System (AIS) and marine AtoN signal lights [1]. 

The development of ports and fairways, together with the recent progresses of technologies and the increasing demands on navigation services make a dynamic optimization of the AtoN systems necessary. The consistency of the AtoN placement will directly influence the safety of marine traffic, so consideration should be given either to the Collision Regulations (COLREG) and local traffic rules.

The knowledge of the weaknesses and problems of the existing AtoN system is of central importance to enhance the provided service. 

In this regard, Chen et al. [2] proposed the Success Degree-Fuzzy comprehensive evaluation method to post evaluate the existing AtoN system to clarify the needs and to point out its optimization. This model works with a series of index, derived in accordance with the AtoN system characteristics and elaborated on the basis of the consideration of users to guarantee its accuracy. The final algorithm allows the valuation of the overall score of the system in a rank from 0 to 100.

The analysis carried out by our team arises from the immediate need of the Venetian institutions and local seafarers to redesign the AtoN system to assure the highest level of safety in the Venice Lagoon. In fact, the installation of the Experimental Electromechanical Module (MOSE or MOdulo Sperimentale Elettromeccanico) at the inlets of the lagoon led to a series of changes in the navigating area (such as the new artificial island at Lido), with remarkable effects on local traffic.

For this reason, we did not make an a priori study on the maritime traffic safety to evaluate the risks of collision or accidents in general as it was not required to proceed with the optimization of the system. We went directly to the problems expressed by the users to find a solution within the shortest possible time: firstly, we analyzed the physical disposition of the AtoN in the lagoon, then we searched for a more flexible solution to the restricted visibility problem which could guarantee the safe conduction of the navigation in any situation. 

We followed a heuristic method which combined IALA, IMO and local normative with the practical needs in order to reach the optimal AtoN disposition: in fact, the iterative and constant update of the system has been one of the key points of the process.

Afterwards, the effective visibility of the signals has been verified using the theoretical considerations given by the IALA Recommendations E-200-2 [3]. The resulting data showed that, although complying with the safety requirements, the light of each AtoN cannot always assure their visibility. 

Basing on E-navigation solutions, an alternative AtoN system has been developed. According to the IMO, E-Navigation can be defined as the harmonized analysis and exchange of marine information by electronic means, with the aim to enhance safety and security of the navigation and protect the marine environment. 

In 2014 the MSC 94 approved the e-Navigation Strategy Implementation Plan (SIP) to complete a list of tasks by 2019. These tasks mainly regard the improvement of the communication systems (in particular the Vessel Traffic Service Portfolio, not limited to the shore stations) and the bridge design and its equipment, to enhance its reliability and resilience and make the interface more user-friendly [4]. This plan, moreover, bases on estimating the effect of e-Navigation applications on reducing navigational accidents, including collisions and groundings of ships falling under the International Convention for the Safety of Life at Sea (SOLAS) [5].

In this context it is worth to emphasize the role played by e-Navigation in relation to the development of Maritime Autonomous Surface Ships (MASS), driverless systems that are increasingly used in many applications (surveillance, research, monitoring, anti-mining, etc.). In this case, the collection of navigational information plays an important role in the decision-making process and can be achieved by collaboration with systems as the AIS [6]. In particular, hazardous scenarios in restricted water, mainly related to collision-avoidance, are identified as one of the main challenging tasks for autonomous navigation systems, which are expected to operate together with conventional vessel thus respecting the COLREG as well [7].

Following the work carried out by the IMO we identified the potential e-Navigation solution: the resulting approach uses the AIS to virtualize the AtoN by the transmission of a coded message containing the required information: the AtoN does not have to be physically located at sea as it will be shown on appropriate electronic system, usually Electronic Chart Display and Information System (ECDIS). 

The employment of Virtual AtoN has already been tested to mark the entrance to a Traffic Separation Scheme (TSS), provide emergency wreck and obstruction markings and to mark offshore structures. In 2012 the General Lighthouse Authorities (GLA) established a port hand virtual buoy at the Rigg Bank, South of Mew Island at the entrance to Belfast Lough, to warn about a nearby sandbank with a depth of 8.4 m. With the Irish Lights Notice to Mariners No. 03 of 2012 the Authority underlined that the information available to mariners will be dependent on their display system and not all transmitted information may be displayed, so users are always encouraged to keep the system up-to-date. Moreover, mariners are requested to give feedback on their experience of AIS to help the Authority to improve the service checking if the AtoN data is available [8].

Moreover, Wright and Baldauf proposed to supplement physical AtoN with V-AtoN in the many inaccessible, remote and ecologically sensitive regions of the world, like for example the Arctic where ice can collide and displace buoys from locations, the tropics where the AtoN placement could damage coral reefs, and other areas devoid of navigational infrastructure [9]. 

In our case, with reference to IALA Guideline 1081 and IALA Guideline 1062, we decided to use Virtual AtoN (V-AtoN) of safe water to give safe route indications. These AtoN were strategically positioned on the points in which the ship changes course or Waypoints (WP). This information on the route is digitized, so it can be modified and sent according to the needs, thus making this type of AtoN a considerable support to the navigation. This method was first tested in Italy in the Venetian Lagoon to provide indications to help the crew during planning operations [10]. Here, situations of restricted visibility usually led to the closure of ports by the Authorities in order to prevent hazards and risks in general. This E-Navigation solution combines the theoretical consideration with the actual characteristics of the navigation in the Lagoon to improve the safety levels and to allow its proper conduction even in restricted visibility conditions. In fact, the V-AtoN thus obtained can be set as Waypoints on the ECDIS onboard to identify the safe route to the berthing point, giving indications to be followed in any situations which would otherwise prevent the normal conduction of navigation.

This paper outlines the establishment of the optimal AtoN system in the Lagoon of Venice and the consequent study on its effective visibility based on IALA guidelines. It follows an overview of the AIS, then the procedure adopted to establish Virtual AtoN in the Venetian Lagoon is presented. The first theoretical approach is based on the maneuvering characteristics of the ships, opportunely contextualized within the Lagoon, and the related safety requirements. The definitive setting of the system is then given through the statistical analysis of the AIS tracks of ships entering the lagoon recorded by the Coast Guard AIS system. 

This system is still being tested: feedback by the users will help to improve and enhance the service, and future studies based on models or simulations will allow to extend this work to any coastal area having the same necessities, generalizing this work for every situation. 

## 2. Classic AtoN System

To study the optimal AtoN disposition we made a first overview on their characteristics: as already stated, they are standardized among all the IALA members. The only distinction provided by the Maritime Buoyage System applies to lateral signals in relation to the membership of each Country of the IALA A or IALA B system: the countries of Region A use red to indicate the left and green for the starboard while those of Region B do the opposite. Other signals are standardized in terms of destination, shape, color and luminous characteristics. For example, the Safe Water signal indicates an area where navigation is safe. This AtoN has white and red vertical stripes, a red spherical mirage (in case of minor buoys) and a 12-s-period white light with a single 10-s flash.

One of the most important features of the luminous signal is its range, indicating the maximum distance at which the light is perceived. In navigation, the luminous range D in nautical miles is used; it is defined as the maximum distance to which the detection of the light beam is guaranteed (not the light source, still hidden due to the terrestrial curvature), considering the meteorological visibility v and the illuminance required at the observer’s eye or threshold, E_T_. The introduction of some photometric quantities is necessary to identify the illuminance.

From a quantum point of view, the light is characterized by a power *W* in Watt that is function of its own wavelength *λ*: the product of the power for the visibility factor function *V_f_*, expressing the actual perception of light to the human eye, gives the luminous flux *Φ* as in Equation (1):

(1) $$\Phi =V_{f}\;\left(\lambda \right)\;W\left(\lambda \right).$$

The luminous intensity *I* is the quantity of flux measured in a certain direction and is calculated as the ratio between the luminous flux *Φ* and the solid angle *ω* of emission as in Equation (2). It is measured in candles:

(2) $$I=\frac{d\Phi }{d\omega }.$$

The illuminance *E* is the ratio between the luminous flux *Φ* and the area of the surface *A* on which it impacts, see Equation (3). It is measured in lumens on squared meters or lux:

(3) $$E=\frac{d\Phi }{dA}.$$

This illuminance *E* can be expressed as a function of meteorological visibility *v* and the distance to the observer *d* through Allard’s law as in Equation (4):

(4) $$E\left(d\right)=\frac{I}{3.43\times 10^{6}}\frac{0.05^{\frac{d}{v}}}{d^{2}}.$$

**Figure 1:**
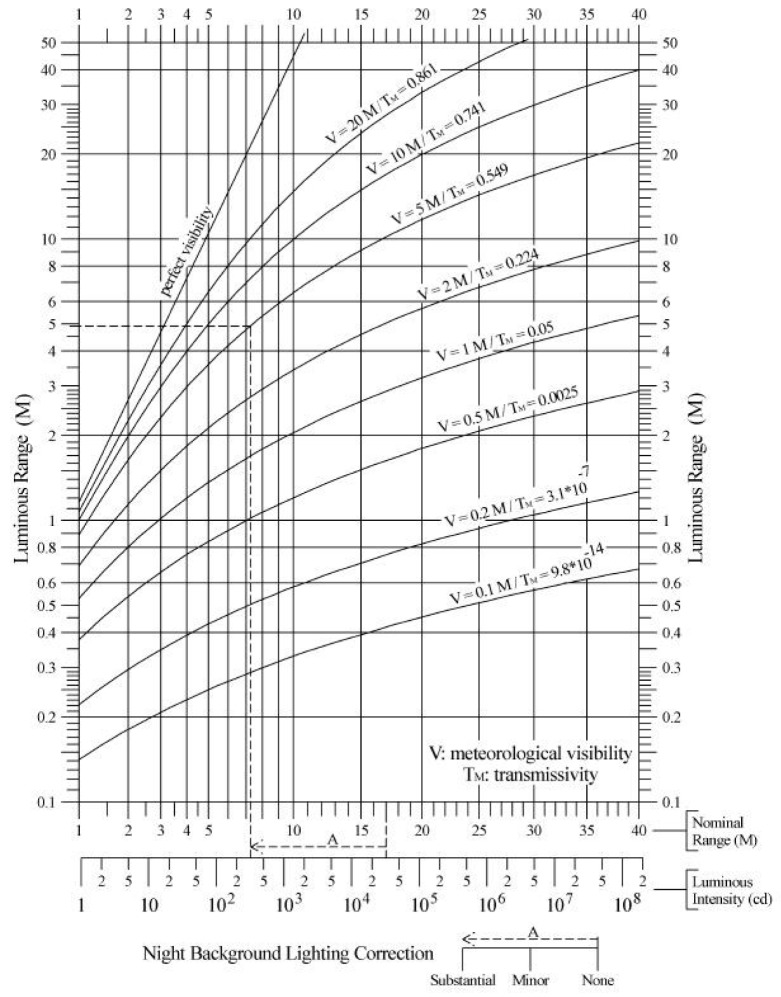
Luminous and nominal range at night.

From Equation (5) it is possible to estimate the luminous range of a signal *D*, which can also be obtained with the diagrams given by the IALA (Figure 1) [3,11,12]:

(5) $$I=\left(3.43\times 10^{6}\right){\;E}_{T}{\;D}^{2}\;0.05^{\frac{d}{v}}.$$

Considering both IALA and local regulations together with seafarers needs, we proposed a maritime signaling system that combines structural efficiency with the highest level of navigational safety for the Venetian Lagoon.

To reach this solution, our team made an accurate survey of the actual AtoN disposition to check the correspondence between the functional AtoN at sea and those reported by the official cartography. This allowed the compilation of an updated database containing all the existing signals with their position and characteristics, according to the IALA standards.

We did not a formal safety diagnosis examination to identify potential safety hazards which may affect the navigation from the initial design phase as done in the Marine Traffic Safety Diagnostic Scheme (MTSDS). In that case, the procedural scheme starts from the analysis of the project and continues with the investigation by a predisposed audit team to review the maritime traffic. After an assessment phase, the audit team will describe all the potential safety problems and suggest all the possible measures to eliminate or mitigate these problems. The most important part of the MTSDS is the process of risk assessment, in which several models can be adopted. The main are the Environmental Stress (ES) Model, the IALA Waterway Risk Assessment Program (IWRAP), the Potential Risk Assessment Model (PARK). The first is based on the acceptance criteria of the stress value based on mariners’ perception of safety, evaluating the difficulty arising from restrictions in maneuvering water area due to the traffic congestion. Three indexes are used: the ES_L_ (Environmental value for Land) calculated on the basis of the time to collision (TTC) with any obstacles, the Environmental stress value for Ship (ES_S_) based on the TTC with ships, and the Environmental stress value for Aggregation (ES_A_), combination between the previous two. However, this model reflects the users’ perception of risks so it would not correct some problems. 

The IALA IWRAP quantifies the risks involved with vessel traffic in specific geographical areas by calculating the annual number of collision and grounding in the specified location; the results are similar to the ES model [13]. The PARK model was developed based on the Korean mariner risk perception and calculates the risk through internal elements (characteristics of the vessel) and external elements such as approaching position of each ship, speed and distance between ships [14].

Due to the urgency of a solution, we decided to directly assess the current situation of the signaling system in the Lagoon through an elementary simulation.

Ji-Min Yeo, et al. studied the implementation of an AtoN database including the integrated system design to develop an AtoN simulator system [15]. In the same way, we set an elementary simulation process using Google Earth Pro (v. 7.3.2.5491) as supporting tool. Among the other features, this software allowed the overlap of the Lagoon official bathymetric and cartographic data to the satellite images. We could then import all the AtoN from our database to get an overall view on the area, thus allowing a more intuitive detection of the current issues and the consequent optimization of the entire AtoN system. 

We began with the theoretical analysis of the area, searching for the best solution in line with IALA standards considering the morphology of the canals as well. Having presented this plan to the Authorities, we integrated the actual needs of the seamen together with those of the involved institutions, trying to reach a trade-off between benefits and costs. 

**Figure 2:**
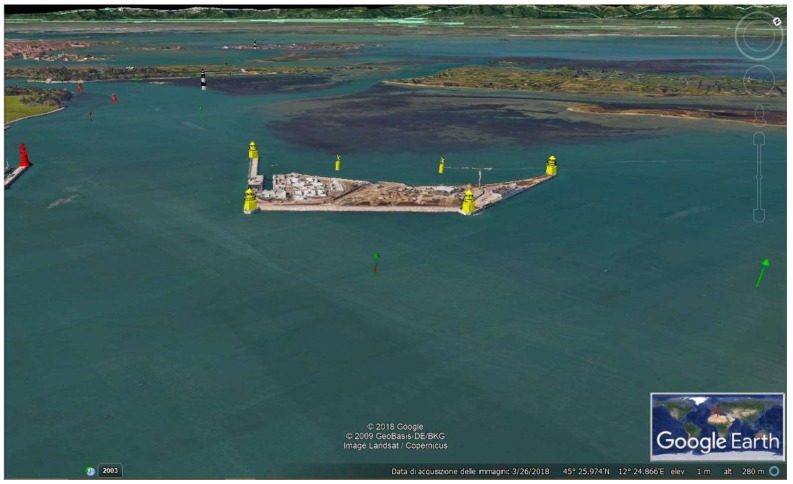
View of the artificial island (Port of Lido). SketchUp models simulate the expected situation.

Some areas needed particular attention. The main examples are the inlets of the Lagoon, where the presence of the MOSE had to be signaled. As previously mentioned, in the specific case of the Port of Lido the artificial island supports the gates, then requires a functional AtoN placement to prevent hazardous conditions. We proposed the installation of yellow flashing lights on the Port entrance lighthouses, to be activated simultaneously to the gates of the MOSE. On the island, four yellow structures could be positioned on its corners; their lights will be red and green for the starboard and the port side of the island respectively (correctly showing the starboard side of the main fairway and port side of the secondary one) and will turn in a yellow flashing light when the barriers are raised, alerting both the units entering and leaving the Lagoon. Since lateral AtoN should be placed in pairs to better indicate the fairway, we assured to the AtoN on the island the correspondent one alongside the canal to identify the main and the secondary canals (Figure 2).

We also proposed some optional improvements, as for example a Preferred Channel signal located before the artificial island to distinguish the main channel on the port side (St. Nicolò Canal) from the secondary one on the starboard side (Treporti Canal). The same has been proposed in the area of Malamocco, where the main fairway turns on the right of the St. Leonardo canal. These buoys are modified Lateral signals: in the first case it will be a horizontally striped green-red-green buoy with a green flashing light while in the second case the stripes will be red-green-red with a red flashing light.

On the basis of the resulting proposals, we used SketchUp 2018 to create a 3D model of each AtoN; then, we imported them on Google Earth to simulate the expected situation in the Lagoon (Figure 2). To verify the efficacy of the proposed AtoN (whose range was given in accordance to those already present) even in poor visibility conditions, we made a purely theoretical analysis based on IALA criteria. The mean distance between each AtoN d and their nominal range D_N_ were known, so we could calculate the intensity produced by each signal as in Equation (5), using the illumination values required by IALA [3]:

(1) *E*_T_ = 2 × 10^−7^ lx at night;
(2) *E*_T_ = 1 × 10^−3^ lx in the daytime hours (to overpower sunlight).

We used the standard visibility of 10 nautical miles, as it is related to the nominal range. From the resulting data of Intensity, we derived the actual illuminance produced by each AtoN as in Equation (4) in three different visibility conditions (obtained from the data recorded by the visibilimeters placed in the Malamocco Canal): perfect, medium and poor visibility. We could then check the effective AtoN visibility as at using Equation (6):

(6) $$E_{d}>E_{T}.$$

As we expected, the results showed the efficacy of the proposal in all the situations except for those of extremely poor visibility, thus making the system not adequate to guarantee the maximum levels of safety in these occasions.

**Figure 3:**
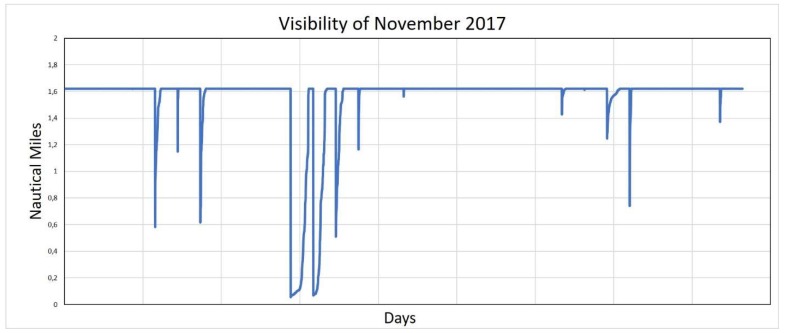
Visibility recorded in the canal of Malamocco in November 2017.

We searched for an innovative tool complementary to the existing AtoN system to manage the problem of the ban on navigation in the Lagoon in case of thick fog: this condition, which frequently occurs in the Lagoon in winter and autumn (Figure 3), do not assure the required safety level according to the Authorities, forcing them to interdict the area and close the ports. 

The increasing development of the E-navigation concept and its related technologies led our team to analyze all the different tools to find a solution which could solve this issue. We identified the AIS as the most reliable and versatile mean to send information to all the units in the working area, thus providing an active service in any situation.

## 3. An Overview on the AIS

Automatic Identification Systems (AISs) and Electronic Chart Display and Information Systems (ECDISs) have become mandatory for ships weighing more than 300 gross tons and for all the passenger ships, irrespective of their size, by the Maritime Safety Committee (MSC) as stated in the International Convention for the Safety of Life at Sea (SOLAS), Regulation V/19.2.4 [16].

The ECDIS is the system authorized by the International Maritime Organization (IMO) to display Electronic Nautical Charts (ENC), that are continuously updated by competent Authorities. This system is supported by the integration with other tools as for example the Global Navigation Satellite Systems (GNSS), Radar and AIS, and shows the details of the ship and the real time area within it is navigating. Unlike traditional charts, the ECDIS displays also bathymetry information and emits an alarm to warn the ship officer about dangerous situations. 

AIS communication systems are Very High Frequency (VHF) radio transceivers that send ship’s data to other units in the working area. Data are collected in real time by the sensors mounted on board and transmitted in broadcast without the need to be operated by ship staff. 

The IMO distinguishes two AIS categories in line with ITU-R M.1371 standards:

1. Class A AIS meets IMO requirements [16,17]. During on-board assembly, the ship’s static characteristics are set (name, dimensions, Maritime Mobile Service Identity-MMSI code, etc.); the AIS is then connected with the onboard GNSS and other related interfaces;
2. Class B AIS units are used by vessels that are not regulated by the IMO, such as leisure craft and fishing vessels. Compared with Class A AIS units, they have reduced functionality and are less powerful. Class B AIS units are designed to co-operate with Class A systems, which is why they do not need to respect IMO performance requirements [18].

### 3.1. AIS Messages

AIS messages consist of a set of digital data packets, for which the ITU defines technical characteristics and global frequencies. AIS Messages, in line with ITU-R M.1371, have a unique identification number from 1 to 27. Standard AIS Messages are divided into four macro information sectors:

1. Static or fixed information, which is set during the on-board installation phase, includes name, destination, dimensions, MMSI code and IMO ID of the ship and the antenna’s position. It is transmitted once every six minutes or when requested by on-shore authorities.
2. Voyage-related information, which is manually entered, updated during the voyage and transmitted every six minutes. It includes the type of hazardous cargo, draught, destination and estimated time of arrival (ETA).
3. Dynamic information is automatically updated from the sensors onboard connected to the AIS. It includes Course Over Ground (COG), Speed Over Ground (SOG), position, time and navigation status.
4. Short safety related messages, broadcast or addressed to a specific unit as required.

AIS units can also transmit a series of messages whose data content is defined by the application: they do not affect the basic operation of the AIS as they do not have the same priority. As a result, secondary information does not cross primary information on the main channels. These messages are known as Application Specific Messages (AIS-ASM) and are generally used to send meteorological communications and warnings in general, but they are not intended to substitute messages sent by standard navigation support services (GNSS, Global Maritime Distress and Safety System-GMDSS, etc.) [19,20].

An example of AIS-ASM is the Route Information Message (ID: Message 8): it can be broadcast or addressed to a specific unit and allows communication of pertinent vessel routing information using waypoints (e.g., mandatory or recommended routes that are not given by official publications). The information has a starting date and time and a duration to respect. As defined by IMO SN.1/Circ. 289, up to five slot messages can be created, but no more than three are recommended, resulting in a maximum of 16 waypoints for each AIS station [19,21]. This limitation was one of the reasons why in this analysis we chose standard messages over AIS-ASM, as explained below. Other specifications can be found in the following documents: IMO SN.1/Circ.289, IMO SN.1/Circ.290, IALA Guideline N° 1028, IALA Recommendation A-124 and annex, Rec. ITU-R M.1371-5.

### 3.2. AIS AtoN

AIS transmitter can also be installed on AtoN to send their position, status and other relevant information, including that provided by ASM, such as meteorological and marine conditions.

The ITU recognizes their potential, as they can work with the VTS and enhance communications in areas where they are installed; the only drawback is the excessive application cost. 

New concepts of AIS AtoN have been then introduced alongside the real one:

1. Synthetic AIS AtoN are physically located at sea but not equipped with a transmitter like the real one. The AIS message, carrying its data and position, is sent by an AIS shore station instead (depending on its range);
2. Virtual AIS AtoN, on the contrary, is not physically at sea: it is only shown in terms of position and other specific information by the AIS Message.

This means that each AIS AtoN message must specify the category to which the AIS AtoN belongs. AIS AtoN can use Message 6, 8 or 14, but the AIS AtoN specific Message has the ID 21: the “Position Report”, in fact, carries all the information needed to locate and recognize the AtoN and its functionalities.

The main identifier for AIS units is the Maritime Mobile Service Identity (MMSI) code; with regards to the Rec. ITU-R M.585-7, this code consists of nine digits, assigned according to the unit (aircrafts, shore stations, etc.). Ships have the following MMSI:

M_1_I_2_D_3_X_4_X_5_X_6_X_7_X_8_X_9_

The first three digits are the Maritime Identification Digits (MID), which denote the country the ship is allocated to (e.g., Italy’s MID is 247). The other digits range from 0 to 9. 

The first two AIS AtoN MMSI digits are always 9 9 and are followed by the MID.

9_1_9_2_M_3_I_4_D_5_X_6_X_7_X_8_X_9_

The sixth digit indicates the category of the AtoN as described below:

1. 99MID**1**XXX, totalling 999 between real and synthetic AIS AtoN;
2. 99MID**6**XXX, for 999 virtual AIS AtoN.

The last three digits also range from 0 to 9 [22]. It is possible to visualize the unit identified by each MMSI code by consulting the ITU Maritime Mobile Access and Retrieval System (MARS) database, daily updated [23].

### 3.3. Virtual AtoN

V-AtoN are an excellent navigation support due to their versatility: being virtual objects, they do not have to be physically placed at sea. They operate through the transmission of a coded digital message sent by authorized systems onshore (the AIS stations, for example) containing the information which is then received by the AIS onboard and displayed on the screen, usually on the ECDIS. 

IALA defines the guidelines on their use and, together with the ITU and the IHO, codifies the technical specifications of the digital messages. V-AtoN approved by the competent authorities play an important role in informing seafarers about visibility conditions, hazards, safe waters, etc.

The interesting feature of this device is the dynamism of the transmitted information, which can be modified depending on the purpose: hence the definition of temporary and permanent Virtual AtoN. While permanent V-AtoN have the same validity as real AtoN, temporary V-AtoN are used to transmit information about temporarily-varying conditions and are sent only when needed. If the temporary use of Virtual AtoN exceeds six months, according to the IHO, it will be considered permanent and shown on the relevant nautical traditional chart [18].

#### Advantages and Disadvantages

V-AtoN messages carry the same information with the same validity as real AtoN, so they are strongly recommended in areas subject to frequent variations, heavily congested routes and critical issues, or where positioning real AtoN could be complex or dangerous.

In fact, V-AtoN can be placed anywhere, regardless of the morphology of the area and with relatively low installation and maintenance costs. Moreover, the on-screen display ensures accurate positioning, while the instantaneous and easy-to-read notifications specific to the area of interest avoids the overload of the system with unnecessary information [24].

However, one of the main issues of this technology is that V-AtoN cannot be received and displayed by all the users. The installation of the AIS in not mandatory for Non-SOLAS unit and vessels under 20 m in length, but they are encouraged to voluntarily fit and use Class B AIS. 

Since it has been designed primarily to provide basic data in a cost effective, reliable and user-friendly product, Class B AIS will not be able to receive and decode the AtoN report contained in the Application Specific Messages or in the AIS Message. So Authorities must be aware that neither the units which do not voluntarily install the Class B AIS nor the units following the recommendation to mount it onboard will have the capability to receive virtual AIS AtoN. 

The different symbology used to depict V-AtoN may cause confusion, although it is expected to be definitively harmonized by 2020. Furthermore, ship officers may be unaware of a V-AtoN thus ignoring their messages or perceiving it as a real AtoN. Therefore, careful consideration is required before replacing a real AtoN with a Virtual one [18]. 

Accordingly to the Irish Authorities, this issue is almost frequent: for this reason, they provide all the necessary details about V-AtoN and their display modality to make all the users able to recognize them on the screen. Summarizing, communications by the users is of essential importance to assess the critical points and enhance the service [8]. 

The dependence of AIS on systems as GNSS should not be underestimated, since it makes the AIS vulnerable to the problems connected to data transmission. In fact, it is not advisable to rely on a single source of information which may be subject to spoofing, intentional interference that misleads the receiver to track counterfeit signals [25].

## 4. Navigate with Virtual AtoN

Assuming that ships usually navigate in the deepest part of the canal, we plotted the safe routes from the inlets of the Lagoon to the inner areas: the coordinates of the resulting Waypoints could have been transmitted as AIS-ASM “Route Information Message”, but associated restrictions made a “change of course” necessary. According to Annex A of IALA Guideline 1081, Safe Water AtoN are recommended as temporary V-AtoN when poor visibility conditions (fog, heavy rain, etc.) prevent the safe navigation of the ship. Considering the safe routes previously plotted, V-AtoN were defined in two different ways described below [24].

### 4.1. The Analytical Method

The first method considers the relationship between the WP coordinates and the ship manoeuvring parameters, together with the geometric route relations [26,27,28]. A 324-m cruise ship has been used in the simulation: according to the Safety Management System (SMS) of its company, the Rate of Turn (ROT) of the ship should not exceed 12 deg/min in confined water (7 deg/min in open water) to ensure passenger comfort. The ROT is the average angular velocity of a ship, measured in deg/min. It depends on its Turning Radius T_R_ and speed V, see Equation (7):

(7) $$T_{R}=\frac{0.955\;V}{ROT}$$

**Figure 4:**
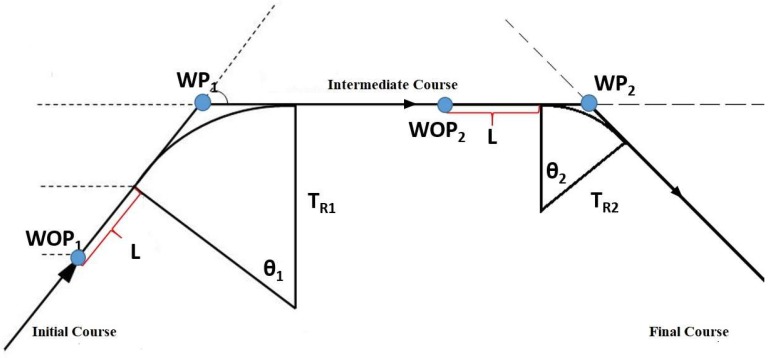
Simplified manoeuvring scheme.

**Figure 5:**
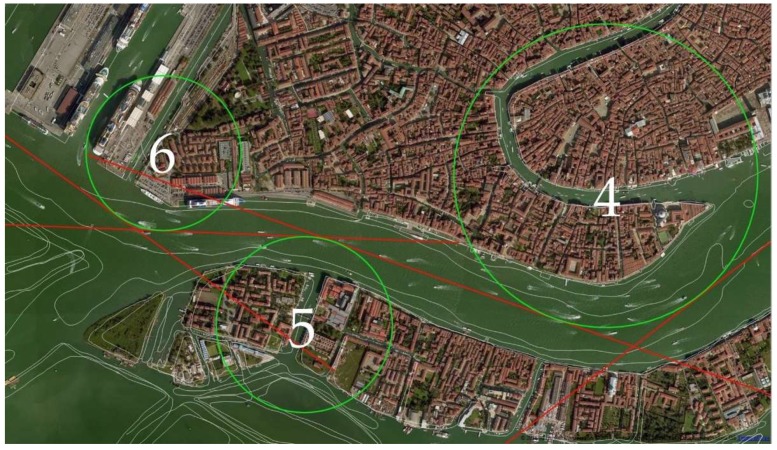
Manoeuvring scheme in the Venetian Canal.

Figure 4 shows a simplified representation of the manoeuvring elements of the ship. We imported the official bathymetric data (white thin lines in Figure 5) provided by the Hydrographic Institute of the Italian Navy into Q-GIS 2.14.15, an open source software, to plot the safe routes in the canals and measure the turning angles *Θ* thus defined (straight red lines in Figure 5). The same has been done for the turning radius *T_R_* of each suitable turning circle corresponding to the manoeuvres, passing through deep and safe water (green circles in Figure 5).

**Figure 6:**
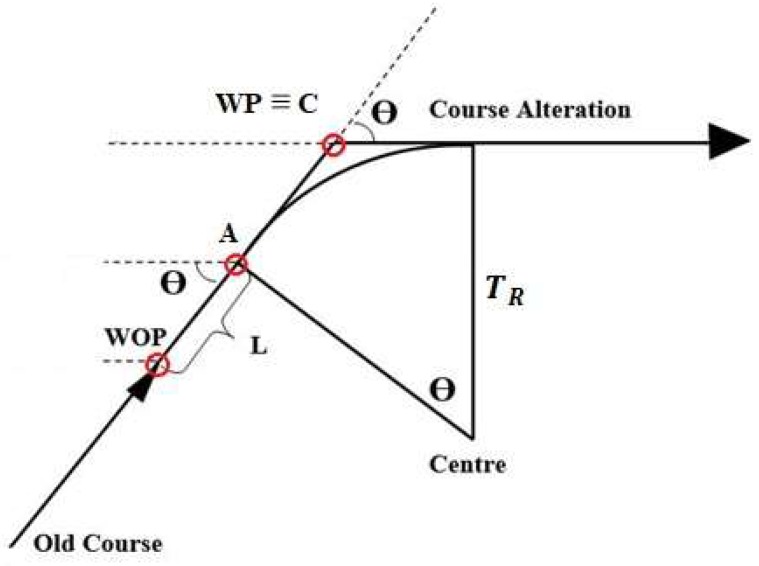
Simplified manoeuvring scheme.

Moreover, since the manoeuvring process of a ship is not instantaneous, the distance on the route between the Wheel Over Point (WOP) and the previous WP should be considered. In correspondence of the WOP the wheel is put over, but only after a distance in general equal to the ship’s length (L) the ship begins to turn. Having identified with *A* this second point and with *C* the WP (Figure 6), the segment AC can be calculated using geometric considerations as shown in Equations (8) and (9).

(8) $$T_{R}\;\left(1-\cos\;\Theta \right)=\bar{\mathrm{AC}}\;\sin\;\Theta ;$$

(9) $$\bar{\mathrm{AC}}=T_{R}\;\tan\;\left(\frac{\Theta }{2}\right).$$

The required distance between WOP and WP is then obtained using Equation (10):

(10) $$\bar{\mathrm{WOP}\_\mathrm{WP}}=\bar{\mathrm{AC}}+\;L.$$

We tested three requirements to position the V-AtoN. 

**Figure 7:**
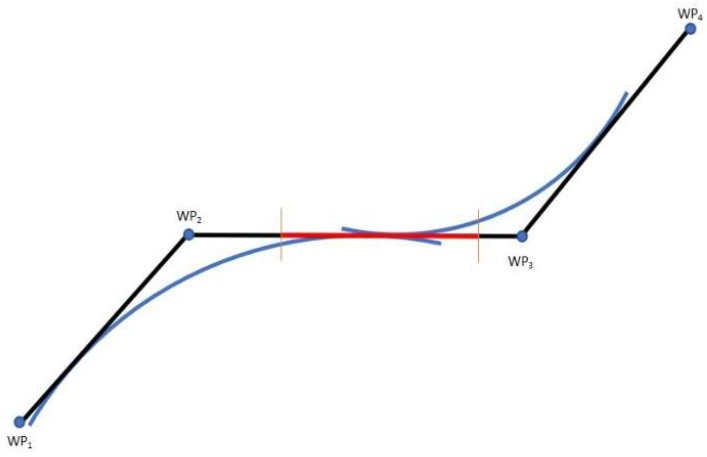
Simplified example of geometrical uncorrected manoeuvre.

The first derives from the relationship between ROT, T_R_ and V, see Equation (7):

1. The maximum speed limit, as stated in the Port of Venice’s Ordinance N° 175/09, is 6 knots in the Lagoon, while the maximum ROT for the ship in question is 12 deg/min. That allows to determine the minimum T_R_ ensuring a safe manoeuvre.
  The second and the third requirements are related to geometrical consideration on the planned route [24]:
2. The sum of the turning radii of two successive manoeuvres (T_R1_ and T_R2_) should not exceed the length of the leg, that is the arc of the great circle (or rhumb line) between two consecutive WP, WP_1__WP_2_ as in Equation (11):
  (11) $$\bar{{\mathrm{WP}}_{1}\_{\mathrm{WP}}_{2}}>T_{R_{1}}+T_{R_{2}}.$$
3. At the same time, the length of the leg WP_1__WP_2_ should allow the ship to safely conclude the first manoeuvre before starting the second one (Figure 7); that means the distance between two following WP (WP_1__WP_2_) should be greater than the distances between WOP and WP related to both the manoeuvres, see Equation (12):
  (12) $$\bar{{\mathrm{WP}}_{1}\_{\mathrm{WP}}_{2}\;}>\bar{{\mathrm{WOP}}_{1}\_{\mathrm{WP}}_{1}}+\bar{{\mathrm{WOP}}_{2}\_{\mathrm{WP}}_{2}}.$$

Going step-by-step through these requirements, we iteratively established the ideal manoeuvring parameters for the simulative ship.

### 4.2. Numerical Approach

Before continuing the analysis, it is important to remember that the ship’s manoeuvrability is significantly affected, amongst other things, by its interaction with the bottom of the waterway; the resulting turning circles will have different characteristics that should then be considered [29]. 

Deep and shallow water are classified according to the ratio between water depth and ship’s mean draught: deep and unrestricted waters have a ratio of at least 3, while shallow waters do not exceed the same value. We set our study and perform the consequent calculus in shallow waters, since the canals in the Venetian Lagoon are not more than 15 m deep and the given ship has a mean draught of 9 m. 

In the first step, we determined the minimum value of T_R_ in the waterways using the relationship defined in Equation (7), valid for each turning circle considering the maximum value of speed and ROT.

$$T_{R_{min}}=\frac{0.955*\;6}{12}\;≅\;0.48\;\mathrm{nm}$$

However, the complex structure of the canals makes it difficult to manoeuvre with these radii, especially if considering the safety requirements previously illustrated. 

An example of the problem is shown in Figure 5 where the three manoeuvres in the Giudecca area (green circles) solely evaluated on the basis of the bathymetry are very closed to each other. In this situation the largest *T_R_*, equal to 0.30 nm, has a value that is shorter than the minimum of 0.48 nm already calculated (Table 1). 

ROT and T_R_ have established values: for this reason, the only way to fulfil the criteria atprovided in Equation (7) is to significantly reduce the speed of the ship; see Equations (13) and (14) respectively for the first and second curve in the Figure 5.

(13) $$V_{1}=\frac{T_{R_{1}}*\mathrm{ROT}}{0.955}=\frac{0.30*12}{0.955}≅\;3.77\;\mathrm{kts}$$

(14) $$V_{2}=\frac{T_{R_{2}}*\;\mathrm{ROT}}{0.955}=\frac{0.18*12}{0.955}\;≅\;2.26\;\mathrm{kts}$$

This implies other issues: in fact, this decrease in speed is unacceptable for a cruise ship since it does not allow to manoeuvre and, in addition, it does not meet the geometrical requirements as the radii are too large. 

**Figure 8:**
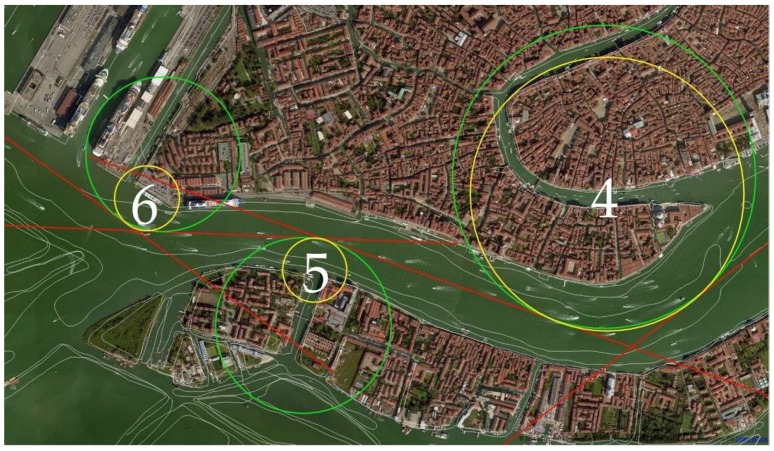
First (green, larger) and last (yellow, smaller) evolution circles in the Venetian Canal.

We used Matlab R2017b (The MathWorks, Inc., Natick, MA, USA) to calculate the ideal manoeuvring parameters for the simulation in question. Starting from the known values of the legs between the WP, the first set of assumed radii and the array of Θ, our code iteratively calculated the radii which best fit the conditions: comparing two following radii, the code decreased the largest one of the 10% of its length, until both finally met the criteria. In our case, 21 iterations were required to achieve an optimal solution which combines all three of the requirements (Table 1, Figure 8).

**Figure 9:**
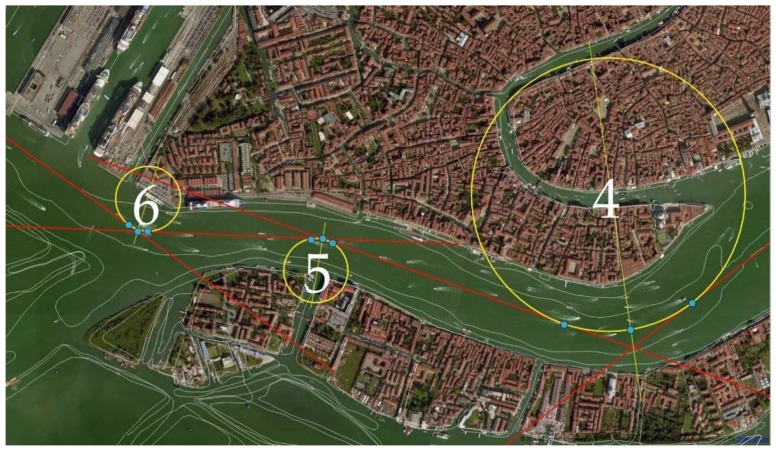
First proposal of Virtual AtoN in the Venetian Canal (light blue dots).

Afterward, we studied the ideal position of the Safe Water Virtual AtoN (Figure 9). We chose three points for each circle: the first and the third on the point of tangency between the circumference and the route (yellow and red respectively), and the second at the intersection of the circle with the segment passing through its centre and the WP (blue dots in Figure 9).

What is important to notify is that all the considerations made to date are based on inaccurate theoretical conditions that do not consider the manoeuvring requirements of the ship. The resulting V-AtoN AIS network, based on fixed geometries and specific evolutionary circumferences, despite complying with the IALA and IMO safety requirements, is then not versatile. In fact, it uses data and information related to the characteristics of a single ship, so the analysed situation and the consequent results are not valid, for example, for a ship with an overall length of 100 m. 

In conclusion, the Virtual AtoN thus obtained cannot be considered a reliable AtoN system for every category of ship. We moved then to a more adaptive solution.

### 4.3. The Statistical Method

We studied the AIS tracks recorded for all the ships navigating in the areas of Lido, Malamocco and Chioggia and we got a solution that combines the simplicity of an easy-to-use service with the enhanced level of safety guaranteed also in adverse weather conditions.

**Figure 10:**
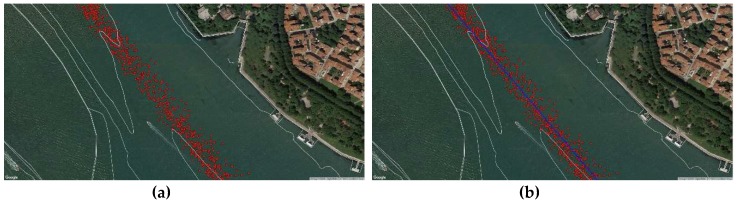
(**a**) Close-up of the AIS position data of ships over 50,000 tons in the Lagoon; (**b**) Resulting statistical followed route.

As previously said, each ship sent its position through the on-board AIS. The coastal station received and stored significant amounts of data, but we selected only those of ships exceeding 50,000 tons, being then able to track their navigation in the Lagoon. We imported this information in Q-GIS (red dots in Figure 10) and estimated the most followed route from the cloud of points using the least-square method.

**Figure 11:**
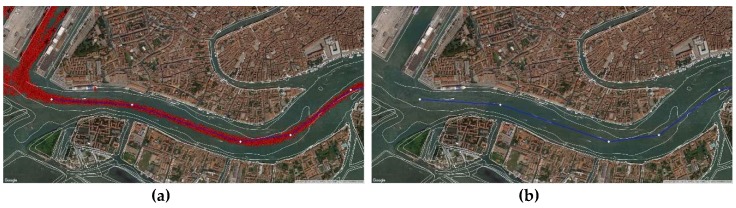
(**a**) Statistical followed route in the area of Venice; (**b**) Resulting Virtual AtoN.

We positioned a V-AtoN on each resulting Waypoint (Figure 11) to define the extremes of safe navigable routes, valid even in poor visibility conditions: displayed on the on-board ECDIS and set as WP, these V-AtoN allow the system to calculate the manoeuvring parameters, taking into consideration the dynamic characteristics of the ship.

**Figure 12:**
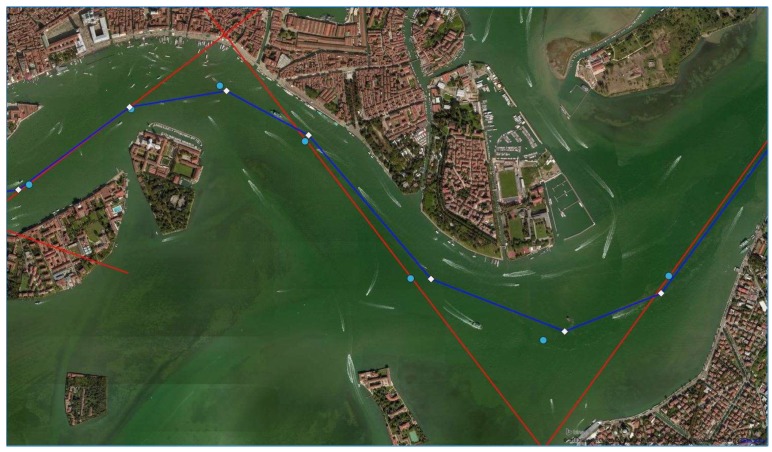
Differences between the WP of the analytical method (light blue dots) and statistical method (white diamonds).

Analysing the WP obtained by both the approaches, we found a maximum difference of 0.1 nm: this is not an excellent result, but it does not compromise the safety of the solution since all the V-AtoN are positioned in safe waters (Figure 12). Hence, they will transmit temporarily AIS Messages when needed, avoiding saturation of the transmission band, and their position will be easily modified according to the variations in width and depth of the waterways, facilitating a safe voyage planning.

## 5. Final Application

As already stated, the conditions of poor visibility that frequently affect the Lagoon of Venice make in this case the navigation not safe enough to be conducted. For this reason, the Authorities are obliged to close the interested area and stop the port operations, resulting in unfavourable situations mainly under the economic aspect. 

The establishment of a system whose operativity is guaranteed in any meteorological conditions is then the solution to this problem. The transmission of the Virtual AtoN WP as previously illustrated helps all the ships to get to the mooring point without the need for the user to worry about the visibility conditions: they will not be able to see the surrounding area, but the information provided by the AIS and displayed on the ECDIS, integrated with the classical aid (as for example the radar) will give all the necessary indications to safely reach the berth, avoiding risks of collisions or grounding. This system can be improved also in relation to autonomous ships, as they can utilise the information provided by V-AtoN (adequately integrated with meteorological and traffic data) to optimize the predictions and the consequent route decisions during navigation.

**Figure 13:**
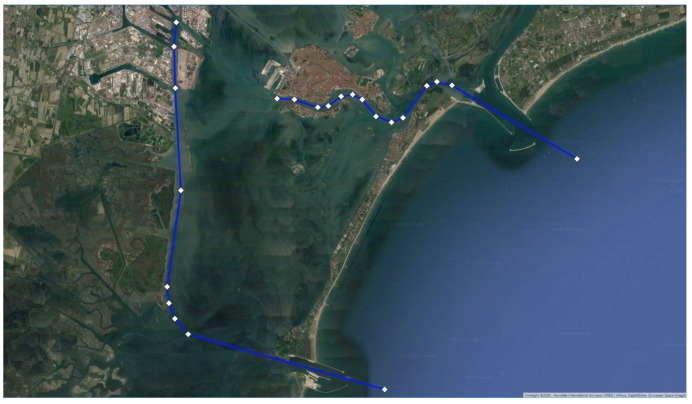
Virtual AtoN for the areas of Lido and Malamocco.

As a result, we defined fourteen V-AtoN in the Giudecca Canal, to allow vessels coming from the Lido inlet to get to the mooring (Figure 13). Similarly, nine V-AtoN have been defined to lead the ships to the Evolution Basin N° 1 in Marghera from the Malamocco inlet (Figure 13). 

**Figure 14:**
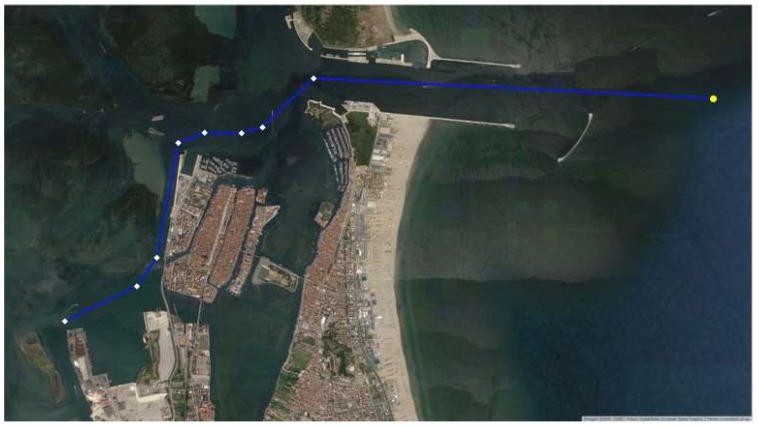
S-AtoN (the first on the right) and V-AtoN in the area of Chioggia.

For the Port of Chioggia, eight V-AtoN were required to plan a safe route to the inner Basin coming from the inlet, where a Synthetic AtoN was placed (Figure 14).

To verify the integrity of the system with respect to the manoeuvring aspect, different enhanced method can be introduced. An example is the Fast-Time Manoeuvring Simulation Technology (FTS) developed at the Institute for Innovative Ship Simulation and Maritime Systems (ISSIMS): it uses a ship-dynamic simulation model to calculate and predict all the manoeuvres the ship can carry out and its motion status. In this way the officer can monitor the manoeuvring actions with the possibility to check for corrections. This new type of support is called Simulation-Augmented Manoeuvring Design and Monitoring (SAMMON) and allows the monitoring of even series of manoeuvring segments [30].

Next step will be the validation of the result. To verify its effective reliability, the Authority of Venice is transmitting the coordinates of the WP as Virtual AtoN every day. The transmission is currently limited to the daily hours to avoid harmful or dangerous situations: in this way, all the users will have the possibility to have a safe approach to this new system and give their considerations with respect to its versatility, user-friendliness and ease of interpretation and, in general, its actual contribution to the navigation during poor visibility conditions. The permanent application of the service will be gradually ultimate once feedback will be received: in fact, the most efficient way to evaluate this kind of solution is to rely on comments by the users to start an optimization process based on their needs. 

The implementation of this E-Navigation system is expected to reduce the number of casualties in the Lagoon improving the safety conditions of the navigation. We did not have precise statistics relative to the traffic conditions in the area, so we could not use a model to assess the effective contribution of the system to the general improvement of the level of safety. From this perspective, further work will be done to overcome this lack of data based on the Marine Traffic Safety Diagnostic Scheme (MTSDS). 

Moreover, it is important to notify that this solution has been essentially structured for SOLAS ships. However, non-SOLAS ships are more vulnerable to accidents, mainly due to the lack of navigational equipment on board leading to less safety information. For this reason, it is necessary to extend this study to all the units. The SMART-navigation approach proposed by Baldaufab and Hong could be used as starting point: it implements the IMO E-Navigation concept including services for non-SOLAS ships. The aim is to identify the units vulnerable to accidents by real time relevant statistics registered by the onshore stations and provide them with additional specific services to prevent the potential accident causes in advance by proactively supporting them. The introduction of new technologies, rules and operational procedures shall be accompanied by adequate training for the staff, so any new application needs to be carefully illustrated to the users [5]. The Virtual AtoN system proposed in this article can be improved and extended to non-SOLAS ships following this line. 

## 6. Conclusions

The analysis carried out in this paper aims to maximize the safety levels of the maritime traffic in any circumstance, in step with the E-Navigation progresses. This derives from the need to guarantee the proper conduction of the navigation even when adverse meteorological conditions affect the normal visibility, mainly during winter and autumn: in this case, the main trouble is the inability to use the classic navigational aids. This scenario is almost frequent in the Lagoon of Venice: here, the only extreme solution to prevent accidents is the closure of the ports by the predisposed Authorities.

Moreover, the installation of the MOSE at the inlets of the Lagoon and the consequent changes in the morphology of the area and in the traffic in general made it necessary an urgent assessment of the actual signaling system. We based our evaluation solely on the needs of the users since we did not have time to simulate different situations with other means. Combining this with the IALA normative and IMO requirements we defined an optimal disposition of the maritime signaling system in the Lagoon; then we checked its effective visibility. Results shown that in case of restricted visibility even this improved AtoN system results inadequate to guarantee a safe conduction of port operations. A different solution was then necessary. 

The recent studies on the E-Navigation concept developed by the IMO and the consequent improvements made on these technologies suggested to deepen their potential to find a reliable and efficient solution to the problem. Studying the various IALA publications on the AIS, we focused on the concept of Virtual AIS AtoN: unlike the Real AIS AtoN, Virtual ones can be placed anywhere, without considering the morphology of the area. In addition, their characteristics can be modified to reflect both the channel and the maritime traffic changes, without high installation and maintenance costs. They can be quickly detected, thanks to the integration between the ECDIS screen and the GNSS instantaneous positioning service; lastly, they can be set as temporary AtoN by transmitting the AIS message only when required, preventing data overload with unnecessary information. Based on the statistical study of the safe routes followed by ships in the Lagoon, we could define a set of maneuvering points, the waypoints, to be sent to the AIS onboard by the predisposed stations onshore. The high number of course alterations needed to reach the mooring from the open sea makes the limit of 16 WP given by the AIS Application Specific Message not enough to use the AIS-ASM method. Therefore, we decided to virtualize and send the WP as Safe Water AtoN. We provided their coordinates to the authorities, which are responsible for authorizing AIS stations to send Message 21 (specific to the AtoN), appropriately set and coded. 

In this way, the on-board AIS will receive a single message containing the AtoN information (including position, type, description), directly displayed on the ECDIS screen. The MMSI code is particularly useful as it provides a unique identifier for each AtoN.

These Virtual AIS AtoN allow the ship officer to detect the recommended route, making a safe navigation possible in any situation. Ship officers should be trained to recognize V-AtoN systems: IALA currently recommends that electronic systems must be used alongside traditional systems, rather than replacing them. 

To maximize the effectiveness of this V-AtoN system, its transmission should be protected: due to its dependence on GNSS, the possibility of spoofing should be considered as it is the AIS major vulnerability. Eventually, the use of application specific messages could be focused to find a solution to the limitations on the number of AtoN to be sent. 

For further improvements, three points should be analysed: the first one is the research of a scientific method to accurately investigate and assess the validity of this kind of solution and its results over the years, following methods of risk assessment. The second one regards the possibility to improve the system with respect to autonomous ships, which could be enhanced thanks to the information provided by the V-AtoN. Finally, the third is related to the availability of the service for both SOLAS and non SOLAS ships; this is of fundamental importance, since units without an AIS system onboard are currently not allowed to take advantage of this V-AtoN system, while it aims to ensure adequate safety levels also to the smallest unit. 

In the meantime, the use of a Virtual AIS AtoN system thus defined represent a valid support to the navigation, being the only safe alternative to closing ports in case of restricted visibility conditions. It is important, however, that they are used in an informed way, adopting all the necessary precautionary actions.

## Figures and Tables

**Table 1:** First and last set of measures in the area of Lido.

	R	T_R_	$\bar{WP_{1}\_WP_{2}}$	$R_{1}+R_{2}>\bar{WP_{1}\_WP_{2}}$	θ	$\bar{AC}$	$\bar{WOP\_WP}$	$\bar{WP_{1}\_WP_{2}}>\bar{WOP_{1}\_WP_{2}}$
	(deg)	(nm)	(nm)		(deg)	(nm)	(nm)	
1	301	0.27	1.61	TRUE	85	0.25	0.42	TRUE
2	216	0.39	1.28	TRUE	106	0.53	0.70	TRUE
3	322	0.33/0.27	0.75	TRUE	89	0.33/0.26	0.50/0.43	FALSE/TRUE
4	233	0.30/0.27	0.66	TRUE	56	0.16/0.14	0.33/0.31	TRUE
5	289	0.18/0.06	0.37	TRUE	17	0.03/0.01	0.20/0.18	FALSE/TRUE
6	272	0.15/0.06			34	0.05/0.02	0.22/0.19	
	306							
